# Reinventing the wheel: a simulation-aided design of a soft, shape-adapting, lugged wheel for locomotion on sandy terrains

**DOI:** 10.3389/frobt.2025.1686519

**Published:** 2025-10-09

**Authors:** H. Shi, P. Klaassen, D. L. Schott, J. Jovanova

**Affiliations:** Department of Maritime and Transport Technology, Faculty of Mechanical Engineering, Delft University of Technology, Delft, Netherlands

**Keywords:** DEM, MBD, shape-adapting wheel, granular terrain, locomotion, simulation-aided design

## Abstract

Locomotion over granular terrain poses significant challenges for autonomous robotic systems, particularly in coastal regions characterized by loose, shifting sands. To optimize the locomotion on these challenging terrains, a simulation-aided design approach was used to develop a soft, shape-adapting, wheeled locomotion system. A co-simulation framework combining the discrete element method (DEM) and multibody dynamics (MBD) is employed to simulate the locomotion of a wheeled robot on varying sandy soils, covering both dry and wet sandy soil conditions. A shape-adapting wheel design is proposed, incorporating soft, inflatable elements that enable the wheel to transform between lugged and circular configurations. A discretized flexbody approach is adopted to model the interactions between the sandy soil and the soft, flexible bodies of the shape-adapting wheel design. Simulation results demonstrate improved performance of the shape-adapting wheels across a variety of sandy terrains, including slopes and obstacles. Integrating softness into the wheel improves obstacle climbing performance, while a lugged wheel configuration performs particularly well on loose, dry sandy slopes. This DEM-MBD co-simulation further enables efficient evaluation of locomotion strategies without the need for extensive physical prototyping.

## 1 Introduction

Locomotion on granular terrain has been a long-standing challenge in both space exploration and terrestrial applications. One of the most iconic examples is the Moon, where lunar soil poses significant difficulties for robotic mobility due to its fine, loosely packed particles. On Earth, desert environments, agricultural fields, and coastal regions represent key examples where robots must navigate loose and shifting substrates. Among these, coastal regions are especially significant. They serve as critical interfaces between land and sea, hosting a wide range of ecosystems, supporting major economic activities and providing habitat for over 40% of the world’s population (United Nations, 2007). Globally, approximately 31% of all coastlines are sandy (Luijendijk et al., 2018), while in the Netherlands approximately 75% of all coastlines consist of sandy beaches and dunes (Giardino et al., 2011).

The exploration and monitoring of these sandy coastal zones have gained importance in a variety of fields, including maintenance and inspection of coastal structures, environmental monitoring and search-and-rescue operations. These tasks are conducted in areas that are difficult or dangerous for humans to access, making autonomous robotic systems a valuable tool. Ensuring that robots can avoid getting immobilized is a critical factor in making autonomous operations practical and safe in these challenging sandy terrains. When sand is compressed under the weight of the robot, the grains lock together and resist deformation. In this state the sand behaves like a solid, offering support and enabling traction. When sand is disturbed by movement of the robot, the grains lose contact and start flowing. In this state the sand behaves like a fluid, offering little resistance and allowing objects to sink into the sand (Li H. et al., 2024; Wang and Wu, 2025). This unpredictable response of sandy soils or the presence of natural obstacles such as rocks, vegetation and slopes can lead to mission failure, especially if the robot becomes immobilized in the sandy terrain.

Robots use various strategies for locomotion on granular terrain. Robotic systems can be classified into six different categories: legged, wheeled, tracked, screw-based, undulatory and vibration-based locomotion, where legged and wheeled locomotion are the most commonly used strategies. Wheeled locomotion strategies are also combined with other locomotion strategies, to create improved systems. The so-called wheel-leg systems combine wheeled locomotion with legged locomotion (Cordes et al., 2018; Shrivastava et al., 2020; Ma et al., 2022), where the wheel is attached at the end of the leg. Wheg designs, which are wheel-like structures with multiple spokes or legs, also combine wheeled locomotion with legged locomotion (Bagheri et al., 2022; Chen et al., 2017; Yun et al., 2017). Wheels with a wave-like surface combine wheeled locomotion with undulatory locomotion (Elsheikh, 2023; Lopez-Arreguin and Montenegro, 2020) and wheel designs with a helical surface combine wheeled locomotion with screw-based locomotion (Lugo et al., 2017; Huang et al., 2022). Various adaptable wheels have also already been developed, such as wheels with a variable diameter (Chen et al., 2011) or extendable lugs (Salazar Luces et al., 2020). Soft robotic locomotion systems have been developed for legged (Liu et al., 2020), snake-like (Li L. et al., 2024) or vibration-based (Kühnel et al., 2016) locomotion systems.

Evaluating the performance of different locomotion systems can be achieved through simulations, eliminating the need for physical prototyping. Therefore, developing a simulation framework to model interactions between robotic structures and sandy terrains is essential for assessing the effectiveness of various design configurations. These interactions are often modelled with terramechanics-based models. Those terramechanics-based models are continuum-based models, which model the granular soil as a continuous medium and rely on empirical relations (Jin et al., 2022). The use of discrete element method (DEM) modelling remains a relatively unexplored approach for evaluating robotic locomotion on sandy terrains. DEM is a numerical, particle-based modelling approach, which represents the granular soil as a finite number of discrete particles, each governed by Newton’s laws of motion (Ravula et al., 2021). The interactions between particles, such as collisions, friction or bonding, are explicitly modelled using contact models, which account for normal and tangential forces, damping and sometimes cohesion. By coupling DEM with multibody dynamics (MBD) simulations, both the robot’s motion and the granular terrain’s behaviour can be accurately captured (Jin et al., 2024; Gao et al., 2024). To model soft, flexible robotic structures, DEM is coupled with multi-flexible-body dynamics (MFBD) simulations, where the DEM part is simulating the granular material and the MFBD part is another co-simulation of MBD and FEM (Zhang et al., 2024a; Zhang et al., 2024b). These types of models would require significant computational power.

To assess the performance of soft robotic locomotion on sandy terrains, this study introduces a novel co-simulation framework that couples the Discrete Element Method (DEM) with Multiflexbody Dynamics (MFBD). Unlike conventional approaches that rely on simplified continuum models or rigid-body assumptions, our framework is uniquely capable of capturing the complex, large-scale deformations of soft, inflatable structures and their interactions with granular soil. This allows for the investigation of how variable stiffness and shape-shifting capabilities influence a robot’s ability to traverse obstacles and adapt to varying terrain conditions, a crucial aspect for off-road robotics. While the simulation models provide a powerful framework for evaluating locomotion performance, a critical limitation has to be noted that they are not a substitute for experimental validation. The idealized conditions of a simulation can fail to capture real-world complexities such as hysteresis in soft materials and dynamic soil compaction. However, the simulation framework should be viewed as a foundational step for initial conceptual designs and it can still capture variations in simplified environmental conditions and provide insights with minimum costs and waste of materials. Therefore, this study consists of both conceptual design evaluations and numerical performance investigations of a soft, wheeled locomotion system, paving the way for future physical prototyping and testing.

Section 2 first presents the simulation framework used to evaluate the performance of robotic locomotion systems. Section 3 presents the design methodology to clarify how simulations are used in the design process. The resulting design of the soft, wheeled locomotion system is presented in Section 4. The performance of the final prototype design is evaluated with simulations, as explained in Section 5. Section 6 presents the conclusions and provides recommendations for future research.

## 2 Simulation framework

**FIGURE 1:**
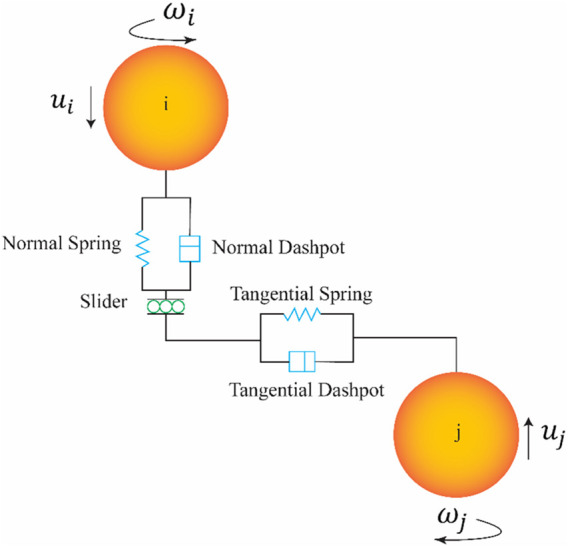
Schematic of the typical contact model used in DEM (Hadi et al., 2024).

A coupled simulation of DEM and MBD is used to simulate the locomotion of a robot on sandy terrains. Altair EDEM 2024 is the used DEM simulation software, and Altair MotionView/MotionSolve 2024 is the used MBD simulation software. The DEM simulation models the granular material and its interactions with equipment. DEM is a numerical modelling approach used to simulate the behaviour of granular materials. DEM represents the granular soil as a finite number of discrete particles, each governed by Newton’s laws of motion. The interactions between particles, such as collisions, friction or bonding, are explicitly modelled using contact models. These contact models account for normal and tangential forces, damping and sometimes cohesion (Ravula et al., 2021). The typical contact model used in DEM is shown in Figure 1. The particle-particle interactions are resolved using a spring-damping system in both normal and tangential direction. The normal and tangential component both include a spring-damper system with stiffness K and damping D , which models how particles resist compression and dissipate energy during collisions. The tangential force computed from the spring damper system is limited by the Coulomb friction law, where the maximum resistive tangential force is determined by the value of the friction coefficient μ .

The multibody dynamics (MBD) simulation predicts the robot’s motion based on the interacting forces and system constraints. A multibody system refers to a collection of interconnected bodies. The bodies in the simulation can be rigid or flexible. A transient analysis is performed to determine how the system responds to loads and movements that change over time. The system responses are displacements, velocities, accelerations and forces, which are calculated using the equations of motion (Schott and Mohajeri, 2023; Schott et al., 2021). Both simulations will run in separate processes, but there is bi-directional communication between the DEM and MBD software. The MBD software will provide at each timestep the positions and velocities of the interacting bodies and EDEM returns the forces exerted by the granular material on the interacting bodies.

**FIGURE 2:**
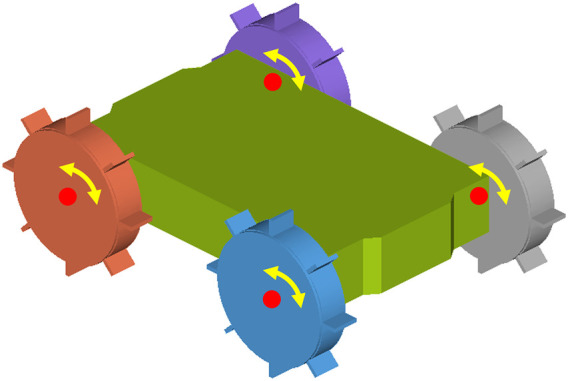
Simulation model of the robot with a rigid chassis and four rigid wheel bodies.

The MBD model of the robot consists of one rigid chassis body and four wheels. Each wheel consists of a rigid wheel hub and a certain number of soft, flexible elements, depending on the wheel design. The rigid wheels are constrained to the chassis body with four revolute joints in the centre of each wheel, which is shown in Figure 2. The motion is applied at each of the four joints, resulting in four-wheel-drive. The type of motion applied to the joints can vary from angular speed, angular acceleration or torque, depending on the goal of the simulation. The chassis of the robot is modelled as a solid body, where it is in reality a shell body with a certain thickness. The extra weight of the solid body compensates for the weight of all the actuation and electronic parts. The timestep of the DEM simulation should be set to a fixed value to ensure that the timestep of the DEM simulation is an exact multiple of the communication interval of the MBD simulation. The fixed value of the timestep in the DEM simulation is set to a value of 2.0e-05 or 2.5e-05 s, corresponding to a Rayleigh percentage of approximately 18%, which is suitable for most of the simulations. However, for the inflated configurations on the loose, dry sand, a lower timestep of 7.0e-06 s ( ≈ 5%) is used to ensure simulation stability.

The next subsection explains the granular, sandy soils that have been used for the simulations. The input parameters related to the materials of the robot bodies for both the DEM and MBD simulations are given in Section 2.2. The modelling of the soft, flexible bodies is explained in Section 2.4.

### 2.1 Sandy soils

Three different sandy soils have been selected, where each soil varies in moisture content. These granular soils are selected from the Soils Starter Pack of Altair EDEM 2024. The non-compressible dry soil is selected to represent dry sand, and the non-compressible sticky soil is selected to represent wet sand. The compressible, sticky soil is selected to represent very wet sand or clay. The input parameters of the three sandy soil types are obtained from the default library of EDEM and are given in Table 1 and the associated contact model was already shown in Figure 1. The moisture content is represented by the cohesion of the soil, which is modelled by the surface energy. Compressibility of the soil is modelled via the plasticity ratio, applicable only to the compressible, sticky, very wet sand. As we are aiming to compare three types of sand, it is essential to keep the basic contact model parameters unchanged across different types of soil, i.e., the coefficient of restitution, static friction and rolling friction are identical. The only differences are surface energy and contact plasticity ratio, which provide a qualitative difference in the soil response, such as the compressibility and stickiness.

**TABLE 1:** Input parameters of the three different sandy soils.

Parameters	Non-compressible dry sand	Non-compressible, sticky, wet sand	Compressible, sticky, very wet sand/clay
Contact model	Hertz-Mindlin (Di Renzo and Di Maio, 2005)	Hertz-Mindlin + JKR (Barthel, 2008)	EEPA (Thakur et al., 2014)
Poisson’s ratio v	0.25	0.25	0.25
Density ρ $\left(kg/m^{3}\right)$	2600	2600	2600
Shear modulus G (MPa)	10	10	10
Co. of restitution e	0.55	0.55	0.55
Co. of static friction $\mu_{s}$	0.2	0.2	0.2
Co. of rolling friction $\mu_{r}$	0.1	0.1	0.1
Surface energy $\gamma_{s}$ $\left(J/m^{2}\right)$	—	3.75	50
Contact plasticity ratio $\lambda_{p}$	—	—	0.7

Upscaled spherical particles are commonly used to model the sandy soils, which minimizes the computational costs (Coetzee, 2017). The size of the sand particles is upscaled to a larger size of 3 mm, because modeling the sand particles with a realistic size of 0.063–2.0 mm would be computationally not feasible. These upscaled particles represent a collection of smaller sand particles, and the parameters are not a one-to-one match for a single sand particle. Therefore, the contact model parameters are also not calibrated against the real sand particles, as it goes beyond the scope of this study. Increasing the particle size by a factor of 1.5 does not influence the main behavior of the granular material (S. Lommen et al., 2019). A particle size of 2 mm has also been considered, but the number of particles would then be around 700,000. When using a 3 mm particle size, the number of particles is around 200,000, which is way better in terms of computational time (from 9 h to 3 h).

### 2.2 Equipment materials

Other input parameters for the DEM simulations are related to the interactions between the equipment materials and the granular material or obstacle. The material properties of the equipment materials and the interaction parameters are all given in Table 2. The robot is designed using two different materials. The soft, flexible parts of the robot are made from silicone rubber. The material properties of standard silicone rubber are used, as the values of the exact silicone rubber material are unknown. The rigid parts of the robot are all 3D-printed from polylactic acid (PLA), which material properties are obtained from (Farah et al., 2016). The interaction properties between the rigid bodies of PLA and the granular soil are set to the default values of Altair EDEM (see Table 2), as these values are hard to find for the specific interactions between sandy soils and PLA. The interaction properties between the silicone soft parts of the robot and the granular soil are obtained from a study on tyre steering on sandy soils (Hu et al., 2021).

**TABLE 2:** Input parameters for the equipment materials and their interactions with the granular material and the obstacle.

Parameters	Standard silicone rubber	Polylactic acid (PLA)
Poisson’s ratio $v_{g}$	0.47	0.36
Density $\rho_{g}$ $\left(kg/m^{3}\right)$	1100	1250
Shear modulus $G_{g}$ (MPa)	20	1287
Co. of restitution $e_{g}$	0.48	0.5
Co. of static friction $\mu_{sg}$	0.55	0.5
Co. of rolling friction $\mu_{rg}$	0.37	0.01
Normal force penalty	500,000	500,000
Normal force co. of restitution	0.1	0.1

For the simulations with the obstacle, a contact is defined between the wheel bodies and the obstacle. The Poisson model is used to model the normal force of the contacts. The penalty of the normal force is set to a high value to reduce the penetration between the bodies and the coefficient of restitution is set to a low value to limit the bouncing (see Table 2). The friction values are set to the same values as for the friction between the particles and the equipment materials. Note that two sets of simulations were performed in current study, one set for concept design selection using component mode synthesis (CMS) technique, and another set using discretized flexbody method to optimised the wheel parameters. The standard silicone rubber parameters were only used for the first set to identify the best concept wheel design choice. While in the second set of simulations, the spring stiffness and damping values were manually calibrated to qualitatively match the behavior of the prototype wheel, which will be elaborated on later.

### 2.3 Modal flexbody representation

**FIGURE 3:**
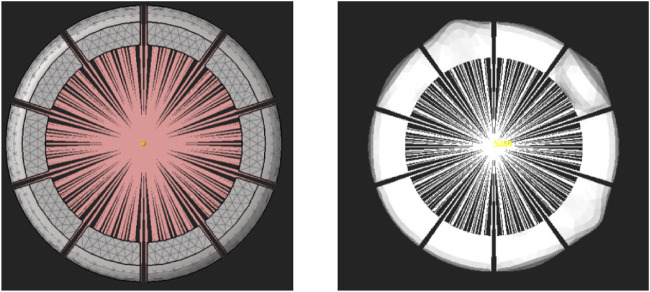
Generation of flexible bodies with the CMS technique: undeformed initial state (left) and deformed state (right).

Modelling the soft, flexible bodies as a finite element model would be too costly in terms of computational time. A more computational efficient method is the component mode synthesis (CMS) technique (Altair Engineering, 2025). In CMS, the displacement of a single element in physical coordinates is represented as a linear combination of a small number of modal coordinates. MotionView uses the component mode synthesis (CMS) technique to create a flexible body. This technique reduces the degrees of freedom of the flexible body to a smaller set of modes. A mode is a specific way in which a structure naturally deforms or vibrates. The computational time for CMS flexible bodies is significantly lower than that of full FEM simulations due to the reduced number of DOFs. While FEM models may involve thousands or even millions of DOFs, a CMS flexible body uses only a limited number of selected modes, 15 in this case. As a result, simulations using CMS flexbodies can be 10 to 1000 times faster than full FEM simulations. However, CMS flexible bodies are only suitable for linear systems, so the deformations of the flexible body have to be small. This method is not ideal for simulating soft silicone parts, as these materials exhibit non-linear behaviour and undergo large deformations. CMS flexible bodies can be generated in MotionView using the built-in FlexPrep tool, which requires a FEM file as the input. The FEM file and the generated flexible body for use in MotionView are shown in Figure 3. The flexible body is connected to the robot with the interface node in the center, which is connected to all the flexible parts via rigid links.

### 2.4 Discretized flexbody representation

**FIGURE 4:**
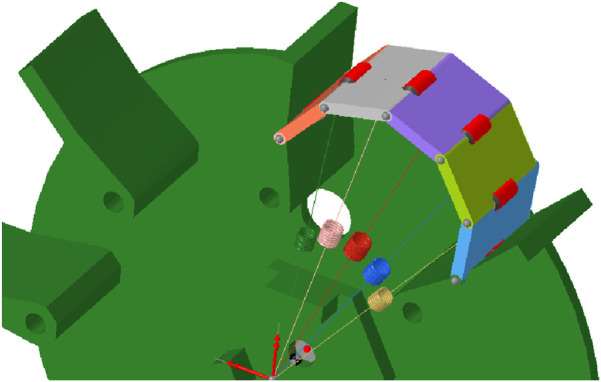
One section of the discretized flexbody representation.

Modeling a soft, flexible robot body as rigid segments connected by springs is a necessary simplification that trades computational efficiency for physical accuracy. A full Finite Element Method (FEM) simulation of a flexible body coupled with a granular DEM model is computationally intensive and would be impractical for large-scale locomotion studies. While it may not accurately predict localized stress or detailed energy dissipation, it is sufficient for analyzing the gross motion and overall kinematic behavior of the robot. Therefore, a simplified approach is used to model the behaviour of the soft, flexible bodies in a more realistic way. These flexible bodies are discretized in multiple smaller bodies, which are connected with bushings to create a chain of bodies (Figure 4). Each small body is also connected to the centre of the wheel with a linear spring-damper, which represents the stiffness of the flexible body. This chain of small bodies can behave like a flexible body and it can undergo large deformations if the spring stiffness is low. In this case the chain of bodies consist of five different bodies to minimize the computational time. The number of bodies will determine the computational time of the simulation, as each extra body will also add an extra bushing and an extra spring-damper. An odd number of bodies is chosen so that the highest point, where initial contact with the surface occurs, is located at the centre of a body rather than at a connection point.

**FIGURE 5:**
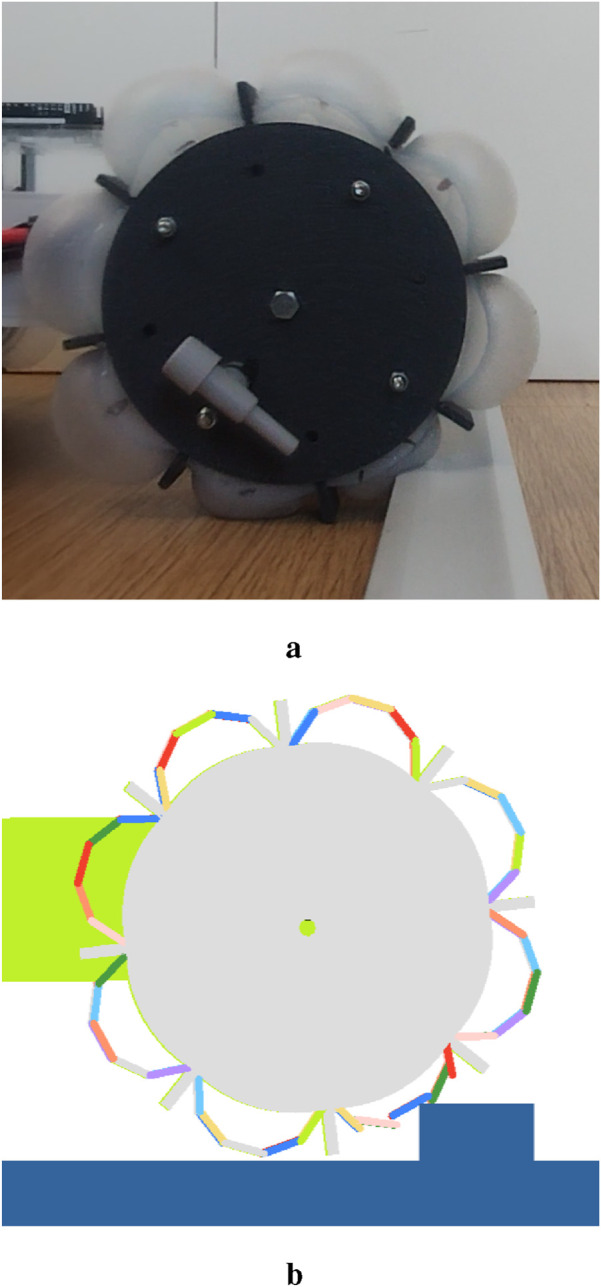
Comparison of the prototype and simulation at a key moment during traversal of a 15 mm high, 30 mm wide obstacle. **(a)** physical prototype **(b)** simulated prototype.

Eight different input parameters are required for the discretized flexbody representation. The bushings require translational and rotational stiffness values in all three directions, as well as corresponding translational and rotational damping values. Additionally, stiffness and damping coefficients have to be assigned for the spring-dampers. These parameters have been obtained by comparing the behaviour in simulations with the behaviour of the physical prototype (see Figure 5), when moving over a small obstacle of 15 mm high and 30 mm wide. The values of spring stiffness and damping were manually tuned to produce the closest qualitative match to the observed behavior (Figure 5a) and are given in Table 3. The stiffness of the whole chain of discrete bodies is not solely dependent on the spring stiffness. The stiffness of the bushings is also playing a significant role and is set to relatively high values, which is required to achieve stable simulations. Low stiffness values would result in high deformations, which would require a smaller timestep to model those deformations. The damping values are also set to high values to reduce the bouncing in the simulation, which enables stable simulations for a higher timestep, and therefore minimizing the computational time.

**TABLE 3:** Input parameters for the discretized flexbody representation.

Simulation parameter	Setting
Bushing translational stiffness (X,Y,Z)	1,000 N/m
Bushing rotational stiffness (X,Y,Z)	1,000 N ⋅ m/rad
Bushing translational damping (X,Y,Z)	15 N ⋅ s/m
Bushing rotational damping (X,Y,Z)	15 N ⋅ m ⋅ s/rad
Spring stiffness #1 & #5	15 N/m
Spring stiffness #2 & #4	11 N/m
Spring stiffness #3	7 N/m
Spring damping	10 N ⋅ s/m

One limitation of this discretized flexbody representation is the ability to model deformation only in two directions without a full material non-linearity. It is not possible to model the deformation of the soft, flexible bodies in the direction parallel to the wheel axis with this method. However, the deformation in this direction is not desired for wheel design, so this limitation does not influence the simulation results. An additional disadvantage occurs when using these discretized flexbodies in DEM simulations with small particles. Those particles can go between the rigid wheel and the chain of discrete bodies, which can influence the results and the stability of the simulation. This is solved by decreasing the timestep for loose granular soils to ensure stable simulations. The construction of the simulation model is quite complex. Each body in the chain of discrete bodies must be imported individually into the simulation environment, and the bushings and spring-damper elements must be added and configured manually. As a result, any change in the design requires the construction of an entirely new simulation model from scratch. This low adaptability is not a concern for this research, as this method will only be used to evaluate the performance of one final design configuration.

## 3 Design methodology

**FIGURE 6:**
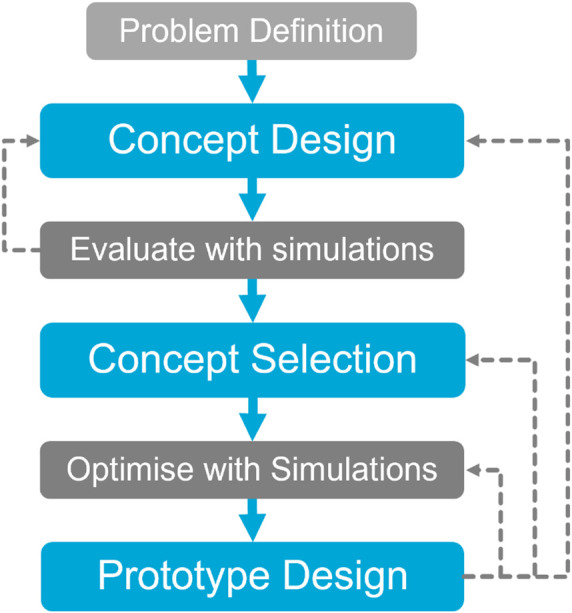
Schematic overview of the design methodology.

**FIGURE 7:**
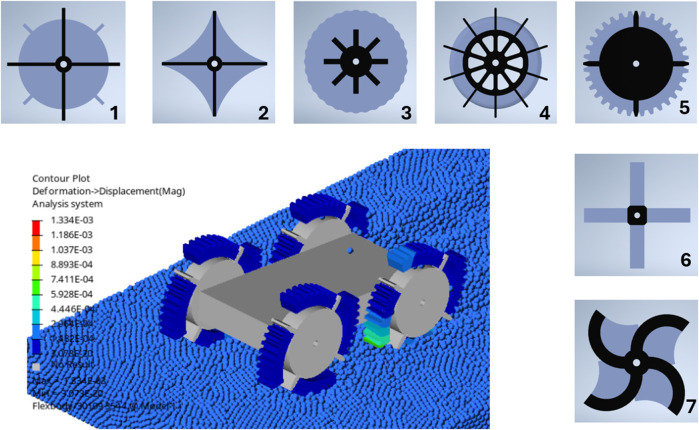
Seven concept designs and example simulation of design five on dry sandy soils using DEM-MBD with CMS technique.

The design methodology of developing a soft, wheeled locomotion method is presented schematically in Figure 6. The design process is supported with simulations. These simulations are used to evaluate the performance of different design configurations. The first design step is the concept design. Seven concept designs are developed to solve the design problem of the locomotion of soft, wheeled structures on dry sandy soils, as shown in Figure 7. The performance of the designed concepts is evaluated with locomotion simulations, where the soft, flexible bodies are represented with the modal flexbody representation (CMS technique). This modal flexbody representation has low computational cost, resulting in the ability to quickly assess the performance of different concept designs.

The selected concept is developed further into the design of a prototype. Several locomotion simulations are conducted to optimize the design of the wheels and the prototype, which is explained in more detail in the following subsections.

### 3.1 Performance evaluation of wheel designs

Five different wheel parameters can be varied to create the optimal wheel design for locomotion on sandy soils: wheel thickness, wheel diameter, lug length, lug thickness and lug spacing.

**FIGURE 8:**
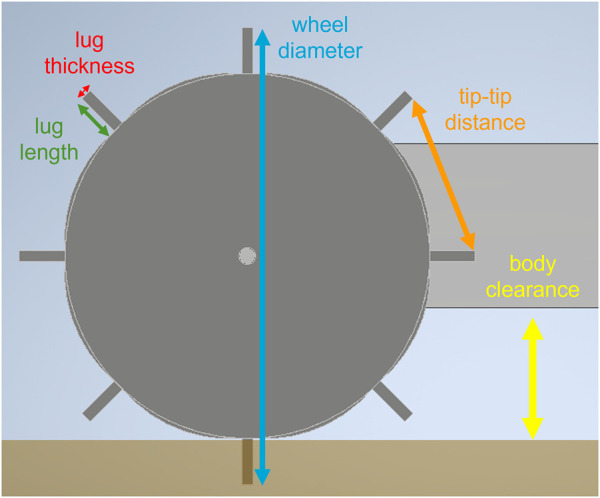
Schematic of the wheel parameters and the robot’s body clearance.

The wheel diameter is indicated with the blue arrow in Figure 8, and is an important parameter for the performance of the robot. Bigger wheels means a larger travelling distance for the same speed. A minimum wheel diameter is required to maintain sufficient body clearance and prevent the robot from becoming stuck on an obstacle or from dragging its body on the ground. The body clearance of the robot is the distance between the soil and the bottom of the chassis and is indicated with the yellow arrow in Figure 8. So, the wheel diameter is based on this body clearance and the size of the robot.

The wheel thickness is not indicated in Figure 8, but corresponds to the width of the wheel in lateral direction, perpendicular to the direction of travel. Thicker wheels provide a larger contact area with the ground, resulting in increased friction and improved stability. However, thicker wheels also increase the weight of the robot and therefore influence the performance, especially on slopes. For steep slopes, thinner wheels have better performance due to their lower weight. On small slopes, thicker wheels have better performance, as they can generate more traction (Laîné et al., 2018). Therefore, the wheel thickness is based on the size and weight of the robot.

The lug length is indicated with the green arrow in Figure 8 and is the distance from the top of the lug to the top of the soft body in deflated configuration. Increasing the lug length increases the travelling performance of the robot (Sutoh et al., 2012; Laîné et al., 2018). However, the lug length is also determined by the design of the inflatable elements. When inflated, these elements should protrude beyond the wheel’s surface, meaning the lug length must be less than or equal to the maximum extension achieved through inflation. The soft, inflatable elements will be made of silicone rubber, which can be easily stretched to more than 200%. However, inflating the soft element for a greater inflation distance would also affect the shape of the soft, inflated element. Therefore, the lug length is set to a conservative value to make sure the soft elements can be inflated without interference.

The lug thickness is indicated with the red arrow in Figure 8. Increasing the lug thickness will decrease the tip-tip distance, which is indicated with the orange arrow in Figure 8. The influence of the lug thickness on the wheel performance is not found in any literature. To investigate this, four motion simulations have been performed to investigate four different lug thicknesses: 2 mm, 5 mm, 10 mm and 15 mm. The performance of each configuration is evaluated by one KPI: the total distance travelled. The wheel thickness is then selected based on the outcomes of the simulations. These simulations are simulations with only rigid bodies to minimize the computational cost.

The lug spacing can be indicated with different measures, namely the tip-tip distance (see Figure 8), angle between two lugs or the number of lugs on the whole wheel. The lug spacing has influence on the traction, but also on the ability to traverse obstacles. In general, the smaller the lug spacing, the better the generated traction (Sutoh et al., 2012). However, the tip-tip distance should be at least smaller than the rupture distance, which is dependent on the lug length and the granular material (Sutoh et al., 2012). The soil in front of the lug is pushed, which creates a destructive phase in the soil. The rupture distance is the horizontal distance of the destructive phase of soil and is dependent on the internal friction angle of the soil (Sutoh et al., 2012). A larger tip-tip distance is also beneficial for the traversing of obstacles, as the robot can climb larger obstacles when the tip-tip distance is larger. To investigate the optimal lug spacing for both soil and obstacles, a few simulations of different lug spacings have been performed. Three different configurations are evaluated: wheels with 6, 8 or 10 lugs. Some obstacles, composed of four spheres arranged in a pyramid shape, are added to evaluate the influence of the lug spacing on the obstacle climbing performance. The performance is evaluated using one KPI: the total distance travelled. Again, the simulations use only rigid bodies to minimize the computational cost.

### 3.2 Integrated robot design

The chassis of the robot is completely designed around the selected motors and corresponding electronics. Each wheel is individually driven by a single geared DC motor. The required motor torque is obtained by performing some simulations to measure the maximum torque on the wheels. The torque is estimated for a rotational speed of 3 rad/s ( ≈ 29 RPM) and 15 rad/s ( ≈ 143 RPM), which is set as the maximum speed the robot should achieve. The electronics are all selected based on the specifications of the selected motor. The chassis is designed around the motors and electronics to achieve an optimal, lightweight robot prototype. The battery, which is the heaviest component, will be placed in the middle of the chassis for an optimal weight distribution.

## 4 Design results

The results of the design process are presented in this section. This includes the optimized wheel design, the soft, inflatable elements design and the integrated robot design.

To select the best concept, three main aspects were considered: simulation performance, adaptability, and fabrication feasibility. The simulation results in Table 4 show that Concepts 1, 3, 4, 5, and the inflated version of six have the best travel performance. Concepts 2, 7, and the deflated version of six perform poorly. In terms of adaptability, Concepts 3, 4, and five are highly adaptable to different terrains and obstacles due to their design. Concept 1, with its simple rigid lugs, has low adaptability, while Concepts 2 and 6, despite being adaptable, have poor travel performance. Based on both performance and adaptability, Concepts 3, 4, and five are the strongest candidates. However, fabrication feasibility becomes the deciding factor. Concept 4 is the most practical to build. Concept 3 requires complex internal chambers, and Concept 5 requires very strong materials and faces issues with granular material interfering with the hub. Therefore, Concept 4 is chosen as the best option because it offers a great combination of high travel performance, adaptability through its shape-shifting ability, and a simple, easily fabricable structure.

**TABLE 4:** Total distances travelled for all configurations of each concept design as shown in Figure 7.

Concept no.	Distance deflated (mm)	Distance inflated (mm)
1	536	—
2	475	455
3	590	—
4	527	559
5	638	—
6	432	579
7	440	473

For the selection of the wheel parameters, locomotion simulations have been performed to optimize the lug thickness and the lug spacing. Four different lug thicknesses are evaluated by a simple locomotion simulation. The results, given in Table 5, show that the travelling performance decreases when the lug thickness increases. Therefore, the optimal lug thickness should be minimized, constrained only by the structural strength required to maintain integrity. Therefore, the lug thickness is set to 3 mm to ensure the structural integrity of the lugs.

**TABLE 5:** Total distances travelled for wheels with four different lug thicknesses.

Lug thickness (mm)	Distance travelled (mm)
2	620
5	610
10	580
15	540

For the lug spacing, three different configurations are evaluated. The results, given in Table 6, show that the travelling performance increases with an increasing number of lugs, which is as expected from the literature (Sutoh et al., 2012). The selected number of lugs is 8, which is the best combination of a large tip-tip distance for the performance on obstacles and a high number of lugs for the performance on granular soils.

**TABLE 6:** Total distances travelled for wheels with three different lug spacings.

Number of lugs	Angle between lugs (deg)	Tip-tip distance (mm)	Distance travelled (mm)
6	60	47	710
8	45	35	770
10	36	28	830

**FIGURE 9:**
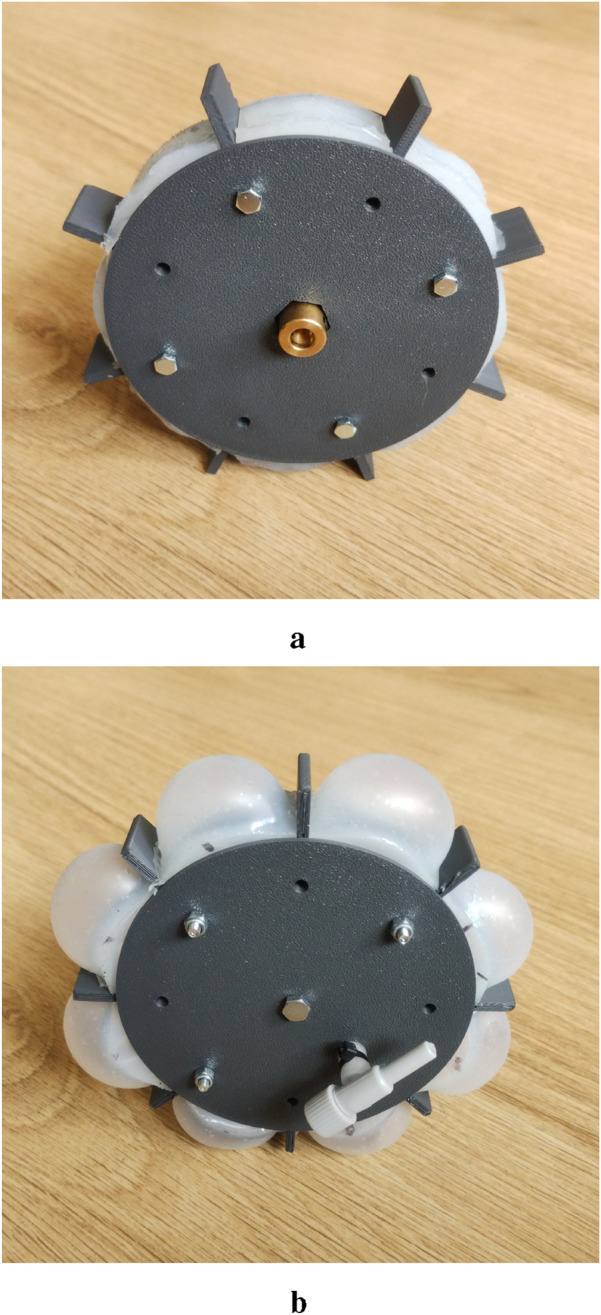
Assembled wheel in inflated and deflated configuration. **(a)** Deflated configuration **(b)** Inflated configuration.

The optimized design of the shape-adapting wheel is shown in Figure 9. The selected values for all five wheel parameters are given in Table 7. The wheel is 3D-printed and consists of two parts which can be connected. The lugs are not connected to the center of the wheel, to leave space for the soft, inflatable elements. With this design, the soft, inflatable elements can be combined into a single soft body, which requires only one air inlet instead of separate air inlets for each element. The soft elements are placed between the lugs and are connected via a ring in the center of the wheel. The two halves of the wheel are fastened with M3 bolts, securing the soft body inside. The soft inflatable element has been fabricated by pouring silicone rubber into a mold. The used material is the Smooth-On ${\text{Ecoflex}}^{TM}$ 00–50 rubber. The ${\text{Ecoflex}}^{TM}$ 00–50 material was selected from the available materials because it has the highest shore hardness (00–50). The air inlet is constructed by attaching a small tube to the outside of the soft part. A small throttle valve is inserted into the small tube to control the air pressure inside the soft part, as shown in Figure 9b. The assembled wheel is inflated by pressurizing the soft part via the throttle valve, which creates the inflated configuration of the wheel shown in Figure 9b. Note that the prototype presented here is mainly used as a conceptual design of a proof of concept; it is not used to perform physical testing, as this goes beyond the scope of the current study.

**TABLE 7:** Overview of the wheel parameters for the optimized wheel design.

Wheel parameter	Value
Wheel diameter	120 mm
Wheel thickness	20 mm
Lug length	12 mm
Lug thickness	3 mm
Lug spacing	8 lugs

**FIGURE 10:**
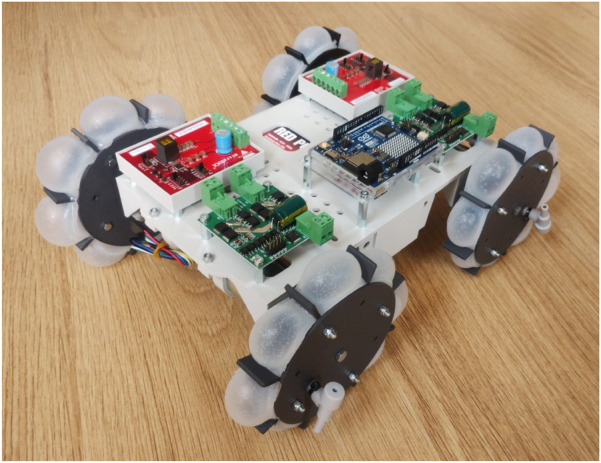
Isometric view of the assembled prototype with inflated wheels.

The centre of the wheel is also connected to the motor via a coupling nut, which is visible in Figure 9a. The chassis of the robot is designed around the motors and electronic components, which results in the total prototype shown in Figure 10. For the motor selection, the required torque has been estimated by using locomotion simulations. The estimated nominal torque is 0.2 Nm and the estimated peak torque is 0.71 Nm. Therefore, a motor with a no-load speed of 200 RPM is required to ensure that the motor generates enough torque at the desired speed of approximately 143 RPM (15 rad/s). The stall torque of the motor should be above 1.0 Nm to ensure that enough torque is available at the desired motor speed. The 12 V geared DC motors are connected to the sides of the chassis. All the electronic components are placed on top of the robot chassis by using spacer nuts to create some air flow for the cooling. The motors are controlled using two dual motor drivers in combination with an Arduino. The input voltage of the motor drivers is regulated by two DC-DC converters. A lithium-ion polymer (LiPo) battery of 14.8 V powers the robot, placed in the middle of the chassis. The total weight of one wheel is around 135 g and the total weight of the prototype around 2.5 kg.

## 5 Prototype locomotion simulations

**FIGURE 11:**
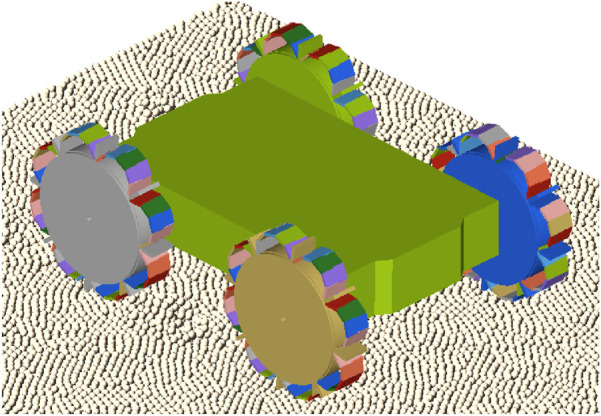
Simulation model of the prototype in inflated configuration.

Using the finalized optimal wheel design in Section 4, the performance of the robot prototype design is evaluated with various simulations of different cases and different soil types. Two configurations of the prototype have been evaluated. For the deflated configuration, shown in Figures 2, 9a, the wheels are modelled as rigid bodies. For this configuration, the soft, inflatable elements will not have any significant influence on the performance, since the granular soil contacts only the rigid sides of the wheel. The inflated configuration, shown in Figures 9b, 11, is simulated by using the discretized flexbody approach.

**FIGURE 12:**
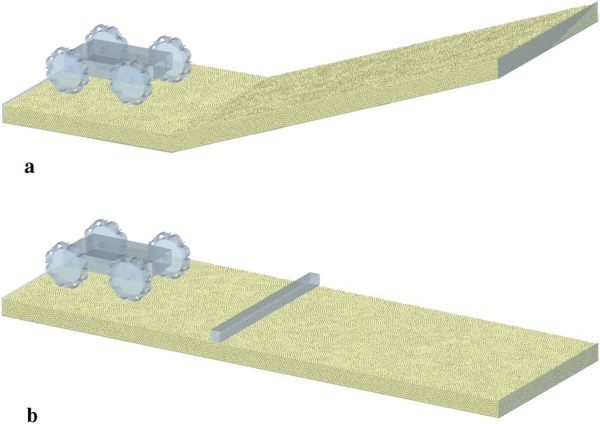
Overview of the two different simulation cases. **(a)** Sloped sand bed **(b)** Flat sand bed with obstacle.

The performance of the prototype is evaluated for two different cases. The first case is evaluating the performance of the prototype when climbing a slope of 20°. The generated sand bed is shown in Figure 12a, where the total length, width and thickness of the sand bed are 1500 mm, 500 mm and 50 mm respectively. The second case is evaluating the performance of the prototype on a flat, sandy surface that includes a rigid 30 × 30 mm square beam obstacle, shown in Figure 12b. The dimensions of the flat sand bed are equal to the dimensions of the sloped sand bed. Both cases are evaluated for two configurations of the prototype across three types of sandy surfaces with varying moisture content. These surfaces are selected to represent different levels of soil cohesion: dry sand (low cohesion), wet sand (medium cohesion), and very wet sand (high cohesion). An overview of all simulations is given in Table 8, where also the timestep of each simulation is given. The motion of the wheels is generated by applying a certain torque, which is dependent on the angular velocity of the wheel, similar to the speed-torque relationship of a DC motor.

**TABLE 8:** Overview of the prototype locomotion simulation results.

Simulation case	Configuration	Granular soil type	Simulation duration (s)	Timestep (s) (Rayleigh %)	Torque (%)	Static sinkage (mm)	Total distance travelled (mm)
Slope	deflated	dry sand	3.0	2.5e-05 ( ≈ 18%)	75	25	754
Slope	inflated	dry sand	3.0	5.0e-06 ( ≈ 5%)	75	13	639
Slope	deflated	wet sand	3.0	2.5e-05 ( ≈ 18%)	50	16	1087
Slope	inflated	wet sand	3.0	2.0e-05 ( ≈ 18%)	50	6	1234
Slope	deflated	very wet sand	3.0	2.5e-05 ( ≈ 18%)	50	20	1170
Slope	inflated	very wet sand	3.0	2.0e-05 ( ≈ 18%)	50	8	1197
Obstacle	deflated	dry sand	2.5	2.5e-05 ( ≈ 18%)	50	24	753
Obstacle	inflated	dry sand	2.5	7.0e-06 ( ≈ 5%)	50	12	891
Obstacle	deflated	wet sand	2.5	2.5e-05 ( ≈ 18%)	50	15	1096
Obstacle	inflated	wet sand	2.5	2.5e-05 ( ≈ 18%)	50	5	1278
Obstacle	deflated	very wet sand	2.5	2.5e-05 ( ≈ 18%)	50	19	1142
Obstacle	inflated	very wet sand	2.5	2.5e-05 ( ≈ 18%)	50	7	1242

Simulations are evaluated using two KPIs: static sinkage and total distance travelled. Static sinkage refers to the vertical displacement of the prototype while it is stationary. Wheel motion begins after 0.5 s, allowing the prototype to sink into the sandy soil, enabling static sinkage to be estimated at 0.5 s. This metric is important, as it affects the prototype’s travelling performance. Less sinkage can enable higher speeds, while greater sinkage may improve traction. The second KPI is the estimation of the total distance travelled by the prototype. This KPI reflects the prototype’s overall locomotion capability and is used to assess how effectively the prototype moves across the sandy terrains.

**FIGURE 13:**
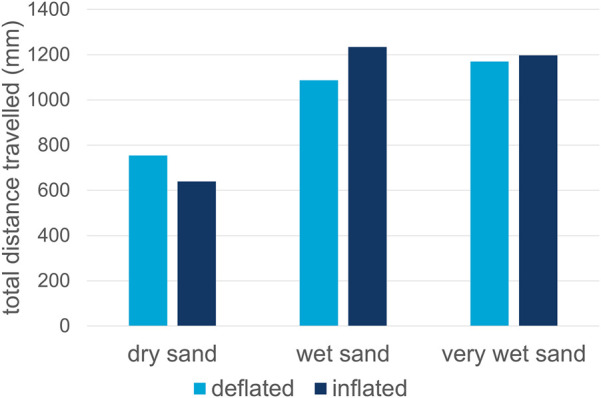
Prototype travelling performance on a sloped surface, simulated for three different sandy soils.

The results of all simulations are presented in Table 8. In general, the sinkage of the inflated configurations is lower than for the deflated configurations. As a consequence, the effective wheel diameter is larger compared with the deflated configuration, which improves the travelling performance of the robot. The total distances travelled for each simulation on a sandy slope are also presented visually in Figure 13. This figure shows the inflated configuration has a better travelling performance than the deflated configuration on more cohesive sandy soils, due to the larger effective wheel diameter and less sinkage. For the loose, dry sand, the travelling performance is worse for the inflated configuration. It is observed that the inflated robot starts slipping in the loose, dry sand when climbing the slope. This is not the case for the deflated robot. Therefore, the deflated robot configuration with the lugged wheel outperforms the inflated configuration on loose, sandy slopes. The inflated configuration enables faster and more efficient locomotion on more cohesive sandy soils, where less sinkage occurs. These observations highlight the value of a variable-stiffness design. A deflated wheel’s larger contact patch reduces pressure on loose, dry sand, preventing sinking and improving traction. Conversely, an inflated wheel’s rigidity allows it to apply concentrated force, effectively gripping the denser, cohesive surface of wet sand. This phenomenon implies that a successful design for all-terrain locomotion should incorporate variable stiffness and shape-shifting capabilities. By adjusting the internal pressure, a robot could optimize its wheel-terrain interaction in real-time. For a practical design, this would involve a control system that uses terrain sensors to autonomously change the wheel’s stiffness, maximizing performance whether on loose, dry sand or a compact, wet surface. This adaptive strategy is a key takeaway for future robotic designs intended for complex and varied environments.

**FIGURE 14:**
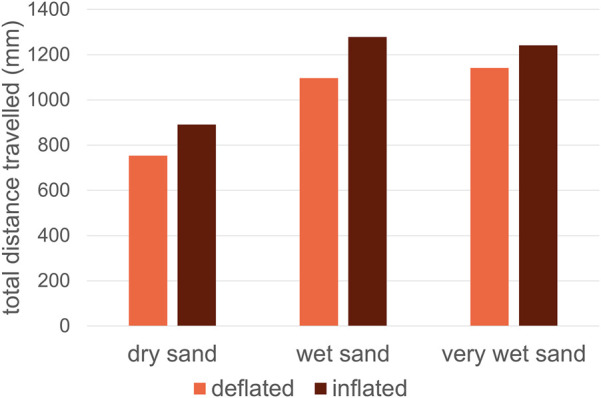
Prototype travelling performance on a flat surface with an obstacle, simulated for three different sandy soils.

For the locomotion simulations with the obstacle, a clear pattern is observed in Figure 14. The inflated configurations are always performing better in travelling a flat sand bed with an obstacle. The total distance travelled for the inflated configurations is higher compared with the deflated configurations. Furthermore, it is observed from the simulation that the inflated configuration tackles the obstacle more smoothly and efficiently than the deflated configuration, resulting in a much more efficient climbing over the obstacle. Therefore, the inflated configuration is always a better choice for traversing obstacles. The soft, inflatable bodies can deform and reshape around the obstacles, providing a strong grip on the obstacles, which enables fast and efficient movements. Even on the loose, dry sandy soil, the inflated configuration still performs better than the deflated configuration.

## 6 Conclusion and recommendations

Capturing the complex interactions between the robot and granular material involves integrating DEM with MBD simulations to simulate the behaviour of both the robot and the granular material. This simulation method is successfully used in the design process to evaluate the performance of different design configurations. A soft, shape-adapting wheel has been designed and a prototype is constructed. The performance of this prototype is evaluated with the locomotion simulations and the results clearly show the benefits of the shape-adapting wheel design. The use of DEM simulations makes it very easy to evaluate the robot performance on various granular terrains, for example on sandy terrains with a varying moisture content.

Various methods for robotic locomotion on granular surfaces are already available in literature. The most used locomotion methods were legged and wheeled locomotion, where a variety of designs already exist. Besides this, tracked, screw-based, undulatory and vibration-based locomotion methods are also possibilities that could be explored. KPIs related to manoeuvrability and traction are the most effective in assessing the travelling capabilities of a locomotion system.

The integration of DEM with MBD simulation is considered the most promising modelling approach for capturing robotic locomotion on granular terrain, as it allows for accurate modelling of both the robot and the granular terrain. The MBD component of the simulation accurately captures the robot’s motion and dynamics, while the DEM component effectively models the behaviour of the granular terrain during interaction with the robot. The soft, flexible bodies can be accurately modelled by using a discretized flexbody representation. This method models the soft, flexible bodies as a chain of multiple rigid bodies, which are connected via bushings. The stiffness and damping is controlled by using multiple spring-damper systems.

The simulation method is used to support the design process. As a result, a shape-adapting wheel is designed, which can be shaped as a lugged wheel and a more circular wheel. Soft, inflatable elements have been placed between the lugs, which can be inflated to change the shape of the wheel. Next to the wheel designs, a robot chassis is designed, which is optimized around the selected motors and electronics, resulting in a lightweight, small robotic prototype with shape-adapting wheels.

The performance of the prototype is only evaluated by simulations for two robot configurations, inflated and deflated, and for two different cases: a sloped sand bed and a flat sand bed including an obstacle. Each of these four different simulations is also evaluated for three different sandy soils: dry sand, wet sand and very wet sand. The interaction between the soft, flexible bodies and the granular soil or obstacles can be captured by the simulation, although it remains a simplified representation of reality, due to the small number of discrete bodies. The performance of the robotic system on exploring sandy terrains with obstacles is significantly better for the inflated wheel, because the soft elements in the wheel enhance the obstacle climbing performance. The deflated, lugged wheel configuration is performing significantly better on loose, dry sandy slopes where a lot of traction is required.

The DEM-MBD framework, while powerful, contains simplifications that affect the reliability of conclusions. For example, the use of idealized particle shapes (e.g., perfect spheres) in the Discrete Element Method (DEM) model does not fully capture the complex interlocking and friction of real, angular sand grains. This can lead to an overestimation of the robot’s mobility and an underestimation of the terrain’s resistance. Similarly, the rigid-body approximations in the Multibody Dynamics (MBD) part of the framework fail to account for high non-linear deformation of the robot’s components under load. This could result in an overestimation of traction and an inaccurate calculation of energy losses. Therefore, the simulation results should be viewed as providing a foundational understanding of the system’s behavior, rather than a direct, quantitative prediction of its real-world performance.

The inflatable design introduces significant practical complexities that go beyond the scope of a simulation. Durability is a major concern, as the soft materials are susceptible to punctures and abrasion, which could compromise the robot’s functionality. Furthermore, a real-world system would require a reliable and energy-efficient system for inflation control, including an onboard pump, pressure sensors, and control algorithms to adjust the robot’s stiffness for different terrains. The energy cost of inflation and deflation, along with the required maintenance procedures for managing air leaks, must also be considered for a successful field deployment. These challenges highlight the need for further research and development to bridge the gap between simulation and a practical, robust robotic system.

Last but not least, a comprehensive evaluation of the proposed simulation-aided framework’s practical application in the real world is beyond the scope of the current study. We acknowledge that our conclusions, which are based solely on simulation assumptions, have limitations without experimental validation. Therefore, it is not possible to fully evaluate the practical realization of these concepts without physical real-world testing. This will also be a critical next step in future research.

## Data Availability

The raw data supporting the conclusions of this article will be made available by the authors, without undue reservation.

## Nomenclature

$\gamma_{s}$: surface energy
μ: coefficient of friction
$\mu_{rg}$: particle-geometry coefficient of rolling friction
$\mu_{r}$: particle-particle coefficient of rolling friction
$\mu_{sg}$: particle-geometry coefficient of static friction
$\mu_{s}$: particle-particle coefficient of static friction
ν: particle Poisson’s ratio
$\nu_{g}$: geometry Poisson’s ratio
ω: motor rotational speed
$\omega_{0}$: motor no-load speed
$\omega_{w}$: wheel angular velocity
ϕ: internal friction angle
ρ: particle density
$\rho_{g}$: geometry density
$\tau_{m}$: motor torque
$\tau_{s}$: motor stall torque
$C_{n}$: damping normal force
$C_{t}$: damping tangential force
$d_{w}$: wheel diameter
e: particle-particle coefficient of restitution
$e_{g}$: particle-geometry coefficient of restitution
G: particle shear modulus
g: gravitational constant
$G_{g}$: geometry shear modulus
$K_{n}$: spring stiffness normal force
$K_{t}$: spring stiffness tangential force
$l_{l}$: lug length
$l_{s}$: rupture distance
$\lambda_{p}$: contact plasticity ratio
**CAD**: Computer-Aided Design
**CMS**: Component Mode Synthesis
**COG**: Centre Of Gravity
**CPR**: Counts Per Revolution
**DC**: Direct Current
**DD**: Dynamic Domain
**DEM**: Discrete Element Method
**DOF**: Degree Of Freedom
**EEPA**: Edinburgh-Elasto-Plastic-Adhesive
**FEM**: Finite Element Method
**JKR**: Johnson-Kendall-Roberts
**KPI**: Key Performance Indicator
**LiPo**: Lithium-ion Polymer
**MBD**: Multibody Dynamics
**PB**: Periodic Boundary
**PLA**: Polyactic Acid
**PWM**: Pulse-Width Modulation
**RFT**: Resistive Force Theory
**RPM**: Revolutions Per Minute
